# Supplementary material for: DDRP: Real-time phenology and climatic suitability modeling of invasive insects
Source: PLoS One. 2020 Dec 31;15(12):e0244005. doi: 10.1371/journal.pone.0244005 (PMC7775054; doi:10.1371/journal.pone.0244005)
Supplement: S1 Appendix — (PDF) [file pone.0244005.s001.pdf]

S1 Appendix. Estimating phenology model parameters for *Epiphyas postvittana*.

The life stage durations used for the DDRP phenology model for *E. postvittana* (light brown apple moth) were estimated using data from a study by Geier and Briese (1980) of the relationship between rate of development and temperature (°C) of immature stages (egg, larvae + pupae) reared on Shorey medium [1] (presented in Fig. 3, p. 138). Using a lower threshold of 7.0°C, this study estimated the duration of the egg, larval, and pupal stage as 131, 421 (female larvae raised on young apple leaves), 510 (female larvae raised on old apple leaves), and 132 degree-days Celsius (DDC), respectively (Table 1).

Data on the oviposition schedule of adult females of two cohorts [1] (presented in Fig. 4, p. 139) were used to estimate the duration of the adult stage, which we defined as emergence of moths through 50% egg laying. One cohort of adult females were raised on Shorey medium and bulk-mated them in sets of eight males and eight females, while the other cohort was reared on broadbean plants and paired individually with a single mate. Moth emergence though 50% oviposition for the two cohorts took an average of 71 DDC (Table 2).

To keep the *E. postvittana* model for DDRP consistent with the one at USPEST.ORG, we rounded the lower developmental temperature threshold presented by Geier and Briese (1980) [1] from 44.6°F to 45°F. We used an empirical conversion to adjust the life stage durations according to this slightly higher threshold. This involved using weather data from 14 U.S. localities (seven for developing the factor, and seven more to test it) where the species is at high risk of establishment (Tables 3 and 4). We used the estimate for egg to 50% egg laying [1] as the generation time.

On average, the percent difference in degree-days when using a lower threshold of 44.6°F versus 45 °F was 97.1% (Table 3). We therefore multiplied life stage durations by 97.1%, which resulted in 229, 734, 231, and 128 degree-days Fahrenheit (DDF) [equal to 127, 408, 128, and 71 DDC; Table 1]. The validation test revealed an average error of 7.2 DDF when using a conversion factor to adjust the duration of egg to 50% egg-laying across the seven validation sites (Table 4).

References

1. Geier PW, Briese DT. The light-brown apple moth, *Epiphyas postvittana* (Walker): a native leafroller fostered by European settlement. In: Kitching PL, Jones RE, editors. The Ecology of Pests: Some Australian Case Histories. Canberra, Australia: CSIRO; 1981. pp. 130–155.

Table 1. Duration of life stages of *E. postvittana* in degree-days Celsius and Fahrenheit based on a lower threshold (Tlow) of 7.0°C (44.6°F) compared to a Tlow of 7.2°C (45°F).

	Tlow = 7°C (44.6°F)		Tlow = 7.2°C (45°F)	
Stage	DDs (°C)	DDs (°F)	DDs (°C)	DDs (°F)
Egg	131	236	127	229
Larva	421	756	408	734
Pupa	132	238	128	231
Adult	74	133	71	128

Table 2. Oviposition schedule of two cohorts of *E. postvittana* in degree-days Celsius based on a lower threshold of 7.0°C (44.6°F).

	Cohort 1	Cohort 2	Average
Time to 5% OV	20	35	28
Time to 50% OV	60	87	74
Time to 75% OV	92	127	110
Time to 95% OV	170	190	180

Table 3. Empirical conversion of the duration of egg to 50% egg-laying (in degree-days Fahrenheit) from a lower threshold (Tlow) of 44.6°F to a Tlow of 45°F.

Replicate	DD (44.6F)	Date	DD (45F)	Percent
Medford OR	1374	06/25/12	1330	96.8
Yakima WA	1373	06/29/12	1335	97.2
Oak Ridge TN	1385	05/03/12	1350	97.4
Salisbury MD	1364	05/21/12	1327	97.3
San Lois Obispo CA	1363	04/28/12	1323	97.1
Salinas CA	1371	05/06/12	1328	96.9
Sacramento CA	1385	05/11/12	1344	97.0
Average	1374		1334	97.1
St. Dev.	8.87		9.82	0.002

Table 4. Validation test of a conversion factor developed to empirically convert the duration of egg to 50% egg-laying (in degree-days Fahrenheit) from a Tlow of 44.6°F to a Tlow of 45°F. Sites used for validation were different from the ones used for developing the conversion factor.

Replicate	DD (44.6F)	Date	DD (45F)	DD (45) estimated	Percent	Error / Absolute error in DDs[a]
Corvallis OR	1365	07/03/12	1317	1326	96.5	9 / 9
Fortuna CA	1370	06/04/12	1320	1330	96.4	10 / 10
Olema Valley CA	1365	06/16/12	1318	1326	96.6	8 / 8
Santa Rosa CA	1365	06/09/12	1320	1326	96.7	6 / 6
Norfolk VA	1377	05/03/12	1339	1337	97.2	-2 / 2
Wilmington NC	1382	04/18/12	1348	1342	97.5	-6 / 6
Hilton Head SC	1372	04/01/12	1339	1332	97.6	-7 / 7
Average	1371		1329	1331	96.9	2.5 / 6.6
St. Dev.	6.67		12.8	6.5	0.005	7.2 / 2.7

[a]The difference in degree-days (Tlow = 45°F) between actual calculated values and values estimated using the conversion factor of 97.1%, developed using the seven sites in Table 3.
