# Supplementary material for: DDRP: Real-time phenology and climatic suitability modeling of invasive insects
Source: PLoS One. 2020 Dec 31;15(12):e0244005. doi: 10.1371/journal.pone.0244005 (PMC7775054; doi:10.1371/journal.pone.0244005)

S2 Appendix. Data sources for validating a DDRP phenology model for *Epiphyas postvittana.*

We used three pheromone trap survey data sets to validate predictions of the dates of first spring egg laying and generation length for light brown apple moth, *E. postvittana*. For each data set, we estimated the date of the peak in first spring flight from bar plots or line plots of moth count data. The first monitoring data set was collected by the U.S. Department of Agriculture's Animal and Plant Health Inspection Service (USDA APHIS) in Alameda, Contra Costa, Monterey, and San Francisco counties in California from 2007 to 2009. Moth count data were reported only on a monthly basis in bar plots, and the precise locations of the study were not reported. Given the coarse spatial and temporal resolution of this data set, we assessed only whether the range of DDRP predictions of first spring egg laying for each county included the month when the observed peak spring flight occurred. We calculated the average, minimum and maximum date of model predictions across the DDRP grid cells for each county.

The second monitoring data set was collected by the University of California Cooperative Extension in and around wholesale nurseries, apples, and berries in five regions of Santa Cruz and Monterey counties in 2012, 2013, and 2014 [1]. Moth count data were collected on a bi-weekly basis and reported in bar plots. We estimated that each region could potentially overlap with four DDRP grid cells (i.e. 8 × 8 km) based on a map of the study regions provided in the report (Fig. 1). We therefore calculated the average, minimum and maximum date of model predictions across the four grid cells for each region. Data for regions in which peak flights were indiscernible were excluded from analyses.

The third monitoring data set was collected by USDA APHIS for a study of population dynamics of *E. postvittana* on four different host plants in Salinas, California in 2019 and 2020. Moth count data were collected on a bi-weekly basis and were available in both raw and line plot format. Peaks in fall and spring flight were virtually identical across the four host plants, so we used single estimates for both. We extracted DDRP model predictions for a single grid cell that overlapped with the location of the study (Fig. 1).

References

1. Tjosvold S. Current Trap Data for Light Brown Apple Moth in Santa Cruz and Monterey Counties. Data: University of California Cooperative Extension, Santa Cruz County [Internet]. Available from: https://ucanr.edu/sites/uccesc/files/157533.pdf

Fig. 1. Map depicting the location of the study areas where the second and third monitoring data sets used to validate *E. postvittana* model predictions were collected. For the second population monitoring data set, we used a map that depicted the location of the five study regions (see pg. 1 of [1]) to estimate which DDRP grid cells each region overlapped with (blue squares). Model predictions for the third data set were extracted from a single grid cell that overlapped with the study location in Salinas (blue triangle). Red lines represent major highways and roads, and grid cells are colored according to the predicted date of first spring egg laying (warmer colors are later dates).

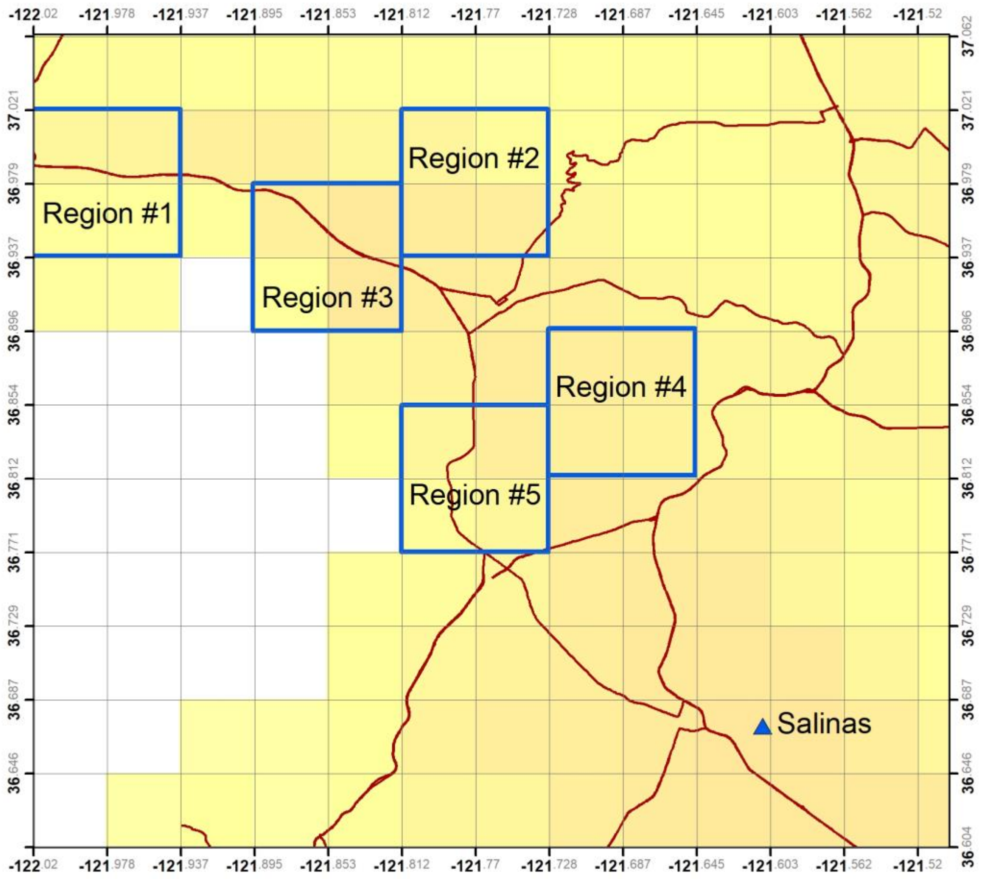

Supplement: S2 Appendix — (PDF) [file pone.0244005.s002.pdf]
