# Supplementary material for: DDRP: Real-time phenology and climatic suitability modeling of invasive insects
Source: PLoS One. 2020 Dec 31;15(12):e0244005. doi: 10.1371/journal.pone.0244005 (PMC7775054; doi:10.1371/journal.pone.0244005)

S3 Appendix. Estimating a common lower temperature threshold and other phenology model parameters for *Neoleucinodes elegantalis.*

Moraes and Foerster (2015) [1] measured the development time of the immature stages of *Neoleucinodes elegantalis* at constant temperatures of 15, 20, 25, 27, and 30°C (50, 59, 68, 77 and 80.6°F; Table 1). They suggested a lower threshold of *ca*. 8.8°C for eggs, 7.7°C for larvae and pupae, and 17.5°C for pre-oviposition. Although large threshold disparities across stages can create problems for simple degree-day models that require a common threshold, the range of thresholds derived from this study was deemed to be a relatively minor issue. The authors did not test cooler temperatures [1], which lowers the accuracy of lower thresholds for each stage. Additionally, only three temperatures were used for measuring pre-oviposition, and the data point for 20°C is not well aligned with the other two points. This result suggests that the estimated lower threshold of 17.5°C may be too high.

We estimated a common lower threshold for *N. elegantalis* using a forced x-intercept method to re-analyze development times reported by Moraes and Foerster (2015) [1]. This involved adding a point to force the x-intercept to a common integer value in degrees Fahrenheit (48°F, 8.89°C) for the egg, egg-to-adult, and pre-oviposition (PreOV) stages (Table 1). Development rates and the development of each life stage in degree-day units were estimated using the regression equation for each stage (Table 2 and Figure 1). The larval and pupal (including prepupae) stage durations in degree-day units were estimated by subtracting the duration of the egg stage from the total egg-to-adult duration and multiplying the resulting value by the average proportion of days to development represented by each stage (Table 3). The summary of the phenology model is reported in Table 4.

In order to test the forced x-intercept approach against alternatives, we compared predictions for egg-to-adult development based on the forced x-intercept model to predictions based on 1) an unforced (i.e. unaltered) model and 2) an unforced model where the lower threshold was simply rounded to 48°F (Table 5). We found that forcing had little impact on predictions (Table 6). The values for the x-intercept and 1/slope for the egg-to-adult interval of *N. elegantalis* without forcing the regression were 8.64°C (47.6°F) and 1048.3, respectively. After constructing the model with forcing, these values were 8.89°C (48.0°F) and 1030.8, respectively. The use of the forced x-intercept method added an average of 1.2 days error compared to the unforced model; however, it had decreased error compared to the unforced model with a rounded threshold (average error = 2.5 days; Table 6).

References

1. Moraes CP, Foerster LA. Thermal requirements, fertility, and number of generations of *Neoleucinodes elegantalis* (Guenée) (Lepidoptera: Crambidae). Neotrop Entomol. 2015;44: 338–344. https://doi.org/10.1007/s13744-015-0293-6

Table 1. Development time in days for *N. elegantalis* reported by Moraes and Foerster (2015). An additional point (in bold font) was added to force the x-intercept to an integer value in degrees Fahrenheit.

	Days Development					
Temp (°C)	Egg	Larvae	Prepupa	Pupa	Egg-to-Adult	PreOV
	**144**				**580**	**164**
15	15.5	40.4	9.1	31.4	96.4	9.8
20	7.3	25.6	4	13.2	50.1	6.5
25	5.3	15.9	3	9.4	33.6	3
27	4.9	16.7	2.6	9.2	33.4	3.8
30	–	13.2	2.1	8.8	–	4.3

Table 2. Development rates, lower threshold (Tlow), and stage durations in degree-days (DDs) for *N. elegantalis* using the modified x-intercept method. An additional point (in bold font) was added to force the x-intercept to an integer value in degrees Fahrenheit.

		Development Rate (1/days)				
	Temp (°F)	Egg	Temp (°F)	Egg-to-Adult	Temp (°F)	PreOV
	**49**	0.0069	**50**	0.0017	**46.6**	0.0061
	59	0.0645	59	0.0104	59	0.1020
	68	0.1370	68	0.0200	68	0.1538
	77	0.1887	77	0.0298	77	0.3333
	80.6	0.2041	80.6	0.0299	80.6	0.2632
	86	–	86	–		–
slope	(b)	0.0064		0.0010		0.0102
intercept	(a)	-0.3083		-0.0466		-0.4878
R^2		0.9948		0.9902		0.9216
Tlow (°F)	(-a/b)	48		48		48
Tlow (°C)	(-a/b)	8.9		8.9		8.9
DDs (°F)	1/slope	156		1031		98
DDs (°C)	1/slope	86		573		55

Table 3. Estimated proportion duration of larval and pupal (including prepupae) stages at three temperatures.

		Days Development				Proportion	
Temp (°C)	Temp (°F)	Larvae	Pupae	Total		Larvae	Pupae
20	59	25.6	17.2	42.8		0.60	0.40
25	68	15.9	12.4	28.3		0.56	0.44
27	77	16.7	11.8	28.5		0.59	0.41
					Average	0.58	0.42

Table 4. Summary of the degree-day model for *N. elegantalis* based on the forced x-intercept method.

	Temp (°C)	Temp (°F)
Lower Threshold	8.89	48
Upper Threshold	32.22	90
Calculation Method	Single Sine	
Model Start	January 1st	

Degree-Day Requirements	DDs (°C)	DDs (°F)
Egg	86	156
Larva + pupa	486	875
Egg-to-Adult	573	1031
Pre-OV	55	98
DDs to Peak OV	95	172
DDs to 90% OV	169	304
Egg-to-1st-OV (min gen. time)	627	1129
Egg-to-Peak-OV (avg gen. time)	668	1203

Events Summary	DDs (°C)	DDs (°F)
First Spring Egg-Laying	55	98
Peak Spring Egg-Laying	95	172
First adults Gen. 1	627	1129
Peak 1st Gen. Egg-Laying	764	1375
Peak 2nd Gen. Egg-Laying	1432	2577
Peak 3rd Gen. Egg-Laying	2100	3780
Peak 4th Gen. Egg-Laying	2768	4983
Peak 5th Gen. Egg-Laying	3436	6186

Table 5. Development rate, lower threshold (Tlow), and duration in degree-days (DDs) for the egg-to-adult stage in *N. elegantalis* estimated using a forced and unforced model.

		Forced	Unforced
Temp (°F)	Days Develop.	1/days	1/days
50	580.0	0.00172	–
59	96.4	0.01037	0.01037
68	50.1	0.01996	0.01996
77	33.6	0.02976	0.02976
80.6	33.4	0.02994	0.02994
slope (b)		0.00097	0.00095
intercept (a)		-0.04657	-0.04537
R^2		0.99022	0.97779
Tlow (°F)		48.00	47.555
Tlow (°C)		8.890	8.642
DDs (°F) (1/slope)		1031	1048
DDs (°C) (1/slope)		573	582

Table 6. Comparison of predicted dates for egg-to-adult development based on a forced model, unforced model, and unforced model with a rounded lower threshold (Tlow). Predictions were generated using three start dates for two generations (without inclusion of pre-oviposition times).

	Forced	Unforced	Unforced with rounded threshold
Tlow (°F)	48	47.56	48
DDs (°F)	1031	1048	1048
Start date: 01/01/20			
1 generation	07/03/20	07/02/20	07/04/20
2 generations	08/22/20	08/20/20	08/23/20
Start date: 04/01/19			
1 generation	07/07/19	07/06/19	07/08/19
2 generations	08/28/19	08/27/19	08/29/19
Start date: 04/01/18			
1 generation	07/08/18	07/07/18	07/09/18
2 generations	08/23/18	08/22/18	08/26/18
Avg. error (days)	1.2	0 (reference)	2.5

Figure 1. Linear regressions of development rates (1/days) for the egg, egg-to-adult, and pre-oviposition (PreOV) stages of *N. elegantalis* at constant temperatures of 50, 59, 68, 77 and 80.6°F (15, 20, 25, 27, and 30°C).

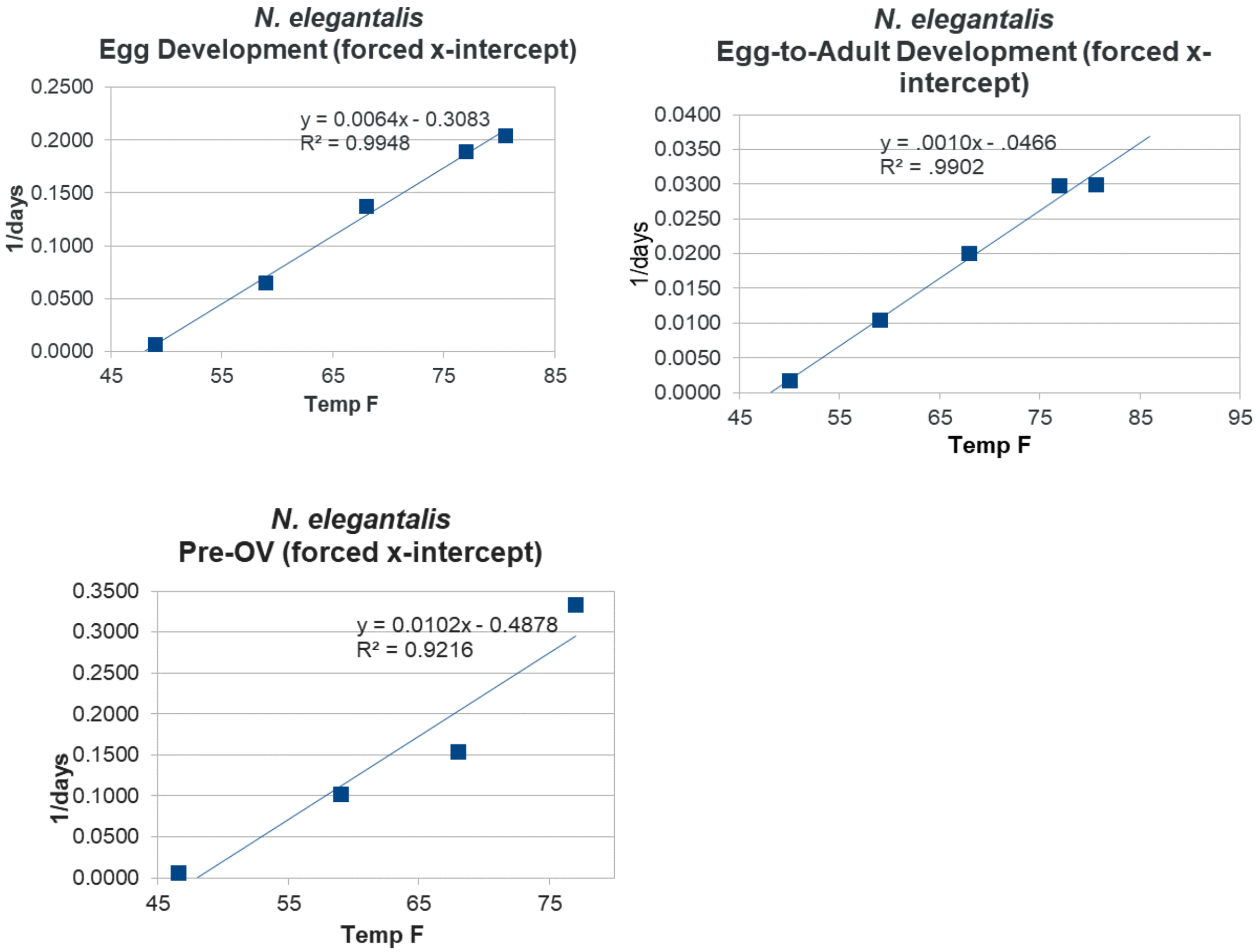

Supplement: S3 Appendix — (PDF) [file pone.0244005.s003.pdf]
