# Supplementary material for: DDRP: Real-time phenology and climatic suitability modeling of invasive insects
Source: PLoS One. 2020 Dec 31;15(12):e0244005. doi: 10.1371/journal.pone.0244005 (PMC7775054; doi:10.1371/journal.pone.0244005)
Supplement: S4 Appendix — (PDF) [file pone.0244005.s004.pdf]

S4 Appendix. Methods for fitting and validating a CLIMEX model for *Neoleucinodes elegantalis*.

To fit and validate a CLIMEX model for *N. elegantalis*, we gathered 181 locality records from GBIF, the literature, graduate theses, and conference abstracts (S1 Table). We randomly subsampled 70% of the records (N = 127) for a model training set and reserved the remaining 30% of records (N = 54) for model validation. We added to the training data set an additional 101 localities that da Silva et al. (2018) used to validate their CLIMEX model for *N. elegantalis* [1], which resulted in a total of 228 localities for model fitting.

Our initial CLIMEX model applied the "best-fit" values presented by da Silva et al. (2018) [1], but this model excluded 22 model training localities from the potential distribution [Ecoclimatic Index (EI) = 0]. In particular, the model appeared to underpredict suitability for *N. elegantalis* in warmer areas of its known distribution. We therefore iteratively adjusted the limiting high temperature (DV3), heat stress temperature threshold (TTHS), and heat stress temperature rate (THHS) parameters and assessed how EI values changed in these areas. All CLIMEX simulations applied a top-up irrigation rate of 2.5 mm day^{-1} for the winter and summer season. We found that the "high" values for these parameters that da Silva et al. (2018) included in their sensitivity analysis (DV3 = 31, TTHS = 31, and THHS = 0.00084) resulted in a good model fit. Specifically, all but six training localities in warmer areas had an EI > 0.

Additionally, we tested different cold stress threshold (TTCS) and cold stress temperature rate (THCS) values. To approximate the lowest temperatures that *N. elegantalis* may be exposed to, we extracted estimates of the historical (1950−2000) minimum temperature of the coldest week at a 10′ minute resolution (Bio6 in the CliMond v1.2 database [2]) for each training locality. The vast majority of localities (225/228 = 98.7%) did not experience historical monthly temperatures lower than 6°C, which suggests that cold stress accumulation at temperatures lower than 6°C may hinder establishment. We applied a TTCS and THCS of 6°C and −0.0005, respectively.

References

1. da Silva RS, Kumar L, Shabani F, Picanço MC. An analysis of sensitivity of CLIMEX parameters in mapping species potential distribution and the broad-scale changes observed with minor variations in parameter values: an investigation using open-field *Solanum lycopersicum* and *Neoleucinodes elegantalis* as an example. Theor Appl Climatol. 2018;132: 135–144. https://doi.org/10.1007/s00704-017-2072

2. Kriticos DJ, Webber BL, Leriche A, Ota N, Macadam I, Bathols J, et al. CliMond: global high-resolution historical and future scenario climate surfaces for bioclimatic modelling. Methods Ecol Evol. 2012;3: 53–64. https://doi.org/10.1111/j.2041-210X.2011.00134.x
