# Supplementary material for: DDRP: Real-time phenology and climatic suitability modeling of invasive insects
Source: PLoS One. 2020 Dec 31;15(12):e0244005. doi: 10.1371/journal.pone.0244005 (PMC7775054; doi:10.1371/journal.pone.0244005)

S1 Fig. Predictions of climatic suitability for *Epiphyas postvittana* in Australia, New Zealand, and California based on 1961–1990 climate normals according to (A, B) CLIMEX and (C) DDRP (California only). Climatic suitability of an area in CLIMEX is represented by the Ecoclimatic Index (EI), where EI = 0 indicates unsuitable conditions. In DDRP, the potential for long-term establishment is indicated by areas not under moderate or severe climate stress. Blue triangles in Australia and New Zealand depict the locations of training locality records used to fit the CLIMEX model (A). Black circles in California depict the locations of records used to validate the CLIMEX and DDRP models (B and C, respectively). CLIMEX maps were generated for this study and have not been previously published.

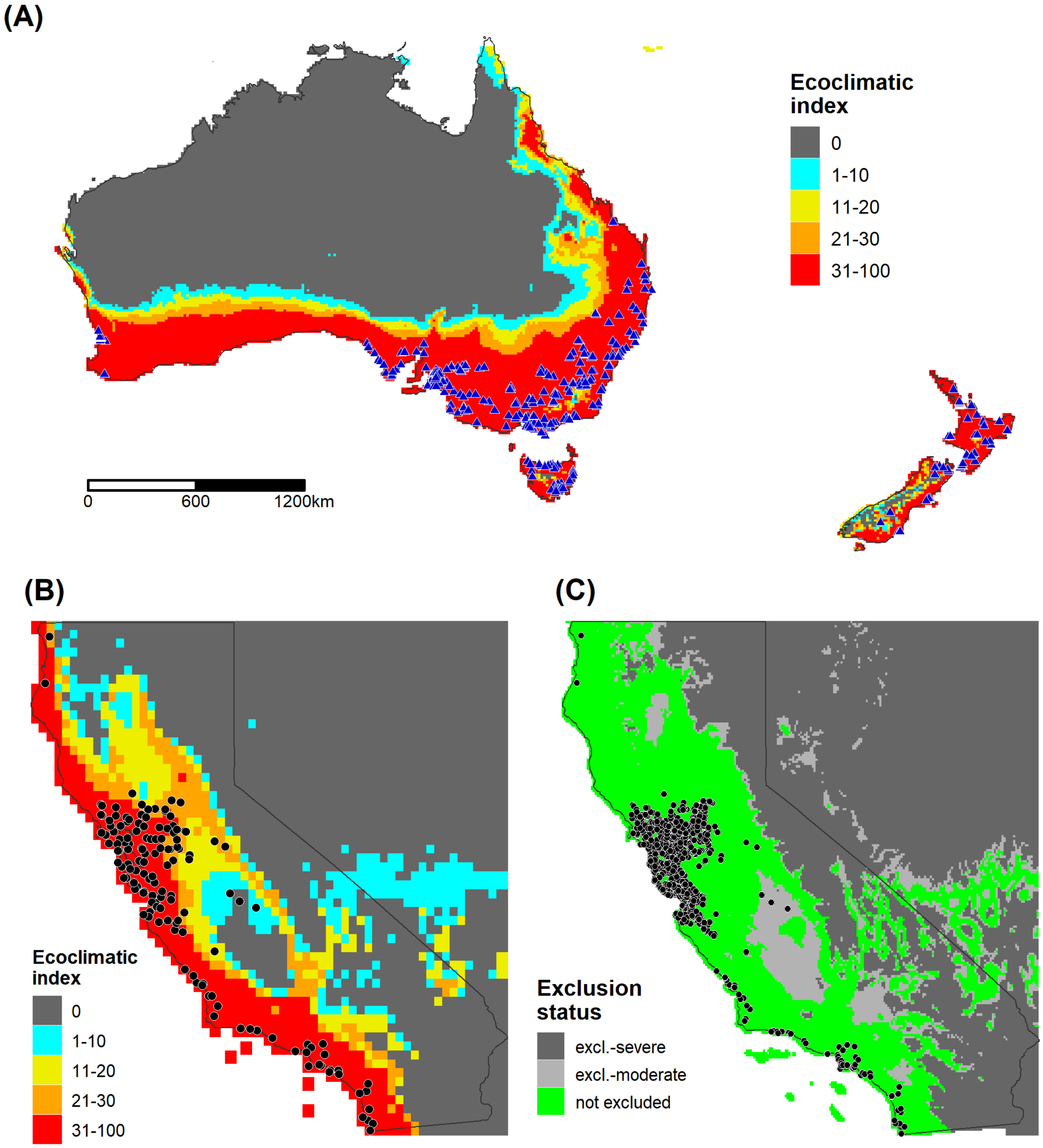

Supplement: S1 Fig — Predictions of climatic suitability for Epiphyas postvittana in Australia, New Zealand, and California based on 1961‒1990 climate normals according to (A, B) CLIMEX and (C) DDRP (California only). (PDF) [file pone.0244005.s005.pdf]
