# Supplementary material for: DDRP: Real-time phenology and climatic suitability modeling of invasive insects
Source: PLoS One. 2020 Dec 31;15(12):e0244005. doi: 10.1371/journal.pone.0244005 (PMC7775054; doi:10.1371/journal.pone.0244005)

S2 Fig. CLIMEX model for *Neoleucinodes elegantalis* in the Neotropics. Climatic suitability of an area is represented by the Ecoclimatic Index (EI), where EI = 0 indicates unsuitable conditions. Blue triangles and black circles depict the approximate locations of occurrence records used to fit and validate the model, respectively. The map was generated for this study and has not been previously published.

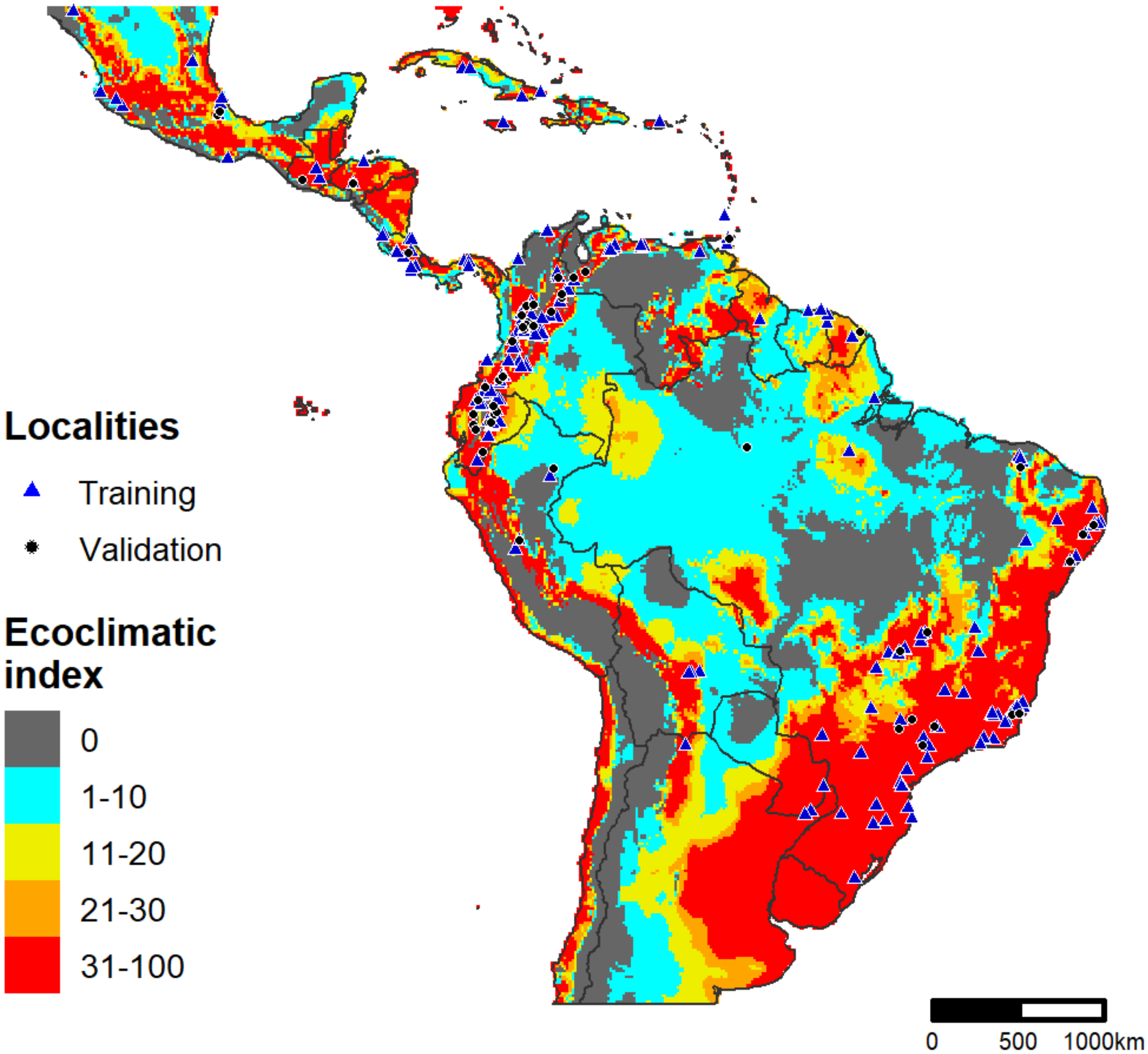

Supplement: S2 Fig — (PDF) [file pone.0244005.s006.pdf]
