# Supplementary material for: DDRP: Real-time phenology and climatic suitability modeling of invasive insects
Source: PLoS One. 2020 Dec 31;15(12):e0244005. doi: 10.1371/journal.pone.0244005 (PMC7775054; doi:10.1371/journal.pone.0244005)

S3 Fig. DDRP predictions of cold and heat stress for (A) *Epiphyas postvittana* and (B) *Neoleucinodes elegantalis* for 2018. Pink and blue lines depict the moderate and severe temperature stress limits, respectively.

(A) *Epiphyas postvittana*

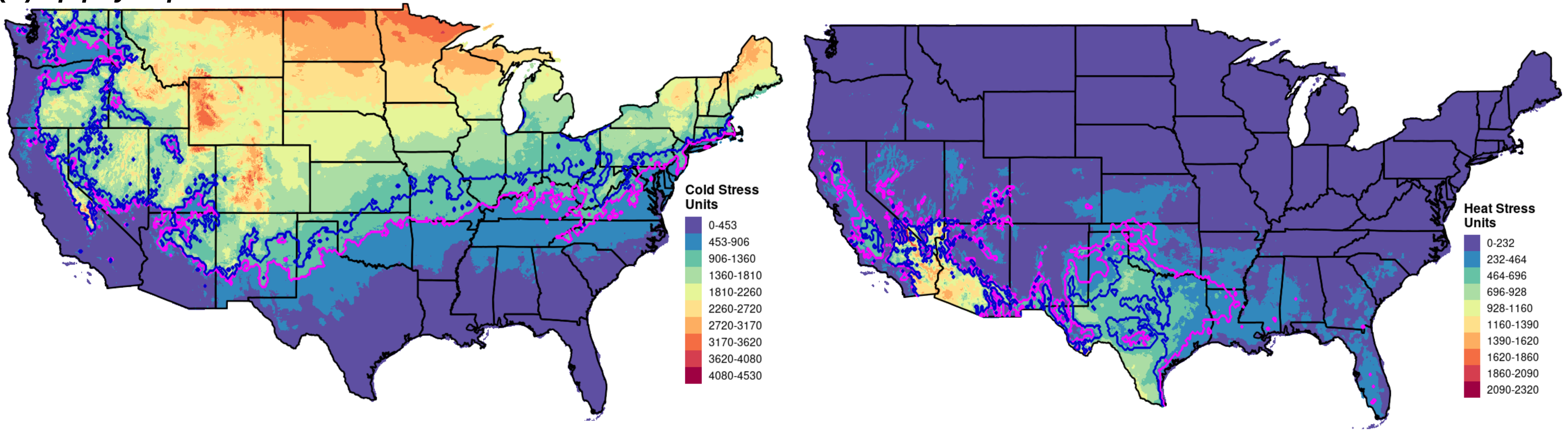

(B) *Neoleucinodes elegantalis*

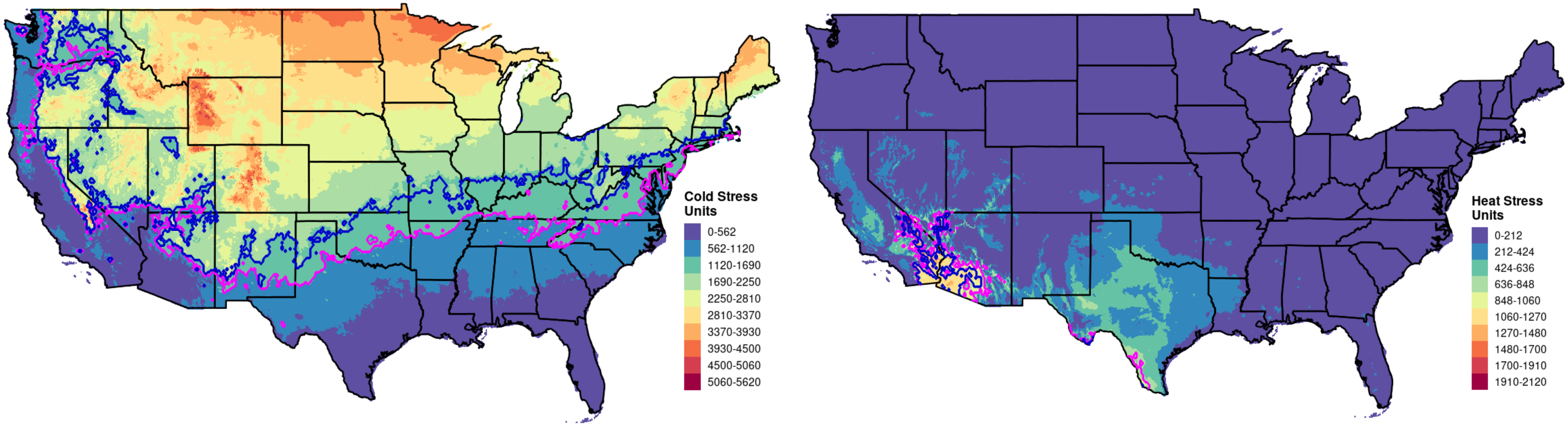

Supplement: S3 Fig — DDRP predictions of cold and heat stress for (A) Epiphyas postvittana and (B) Neoleucinodes elegantalis for 2018. (PDF) [file pone.0244005.s007.pdf]
