# Supplementary figures and images for: DDRP: Real-time phenology and climatic suitability modeling of invasive insects
Source: PLoS One. 2020 Dec 31;15(12):e0244005. doi: 10.1371/journal.pone.0244005 (PMC7775054; doi:10.1371/journal.pone.0244005)

**S4 Fig. CLIMEX predictions of dry stress for *Neoleucinodes elegantalis* in CONUS.**

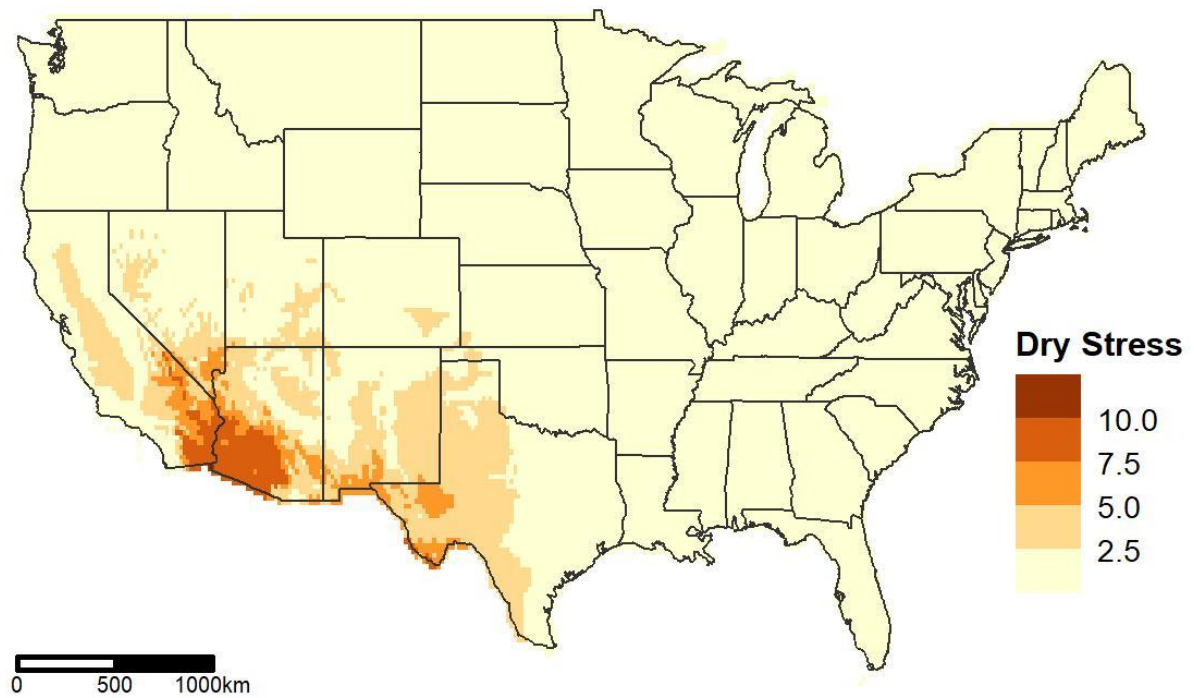

Supplement: S4 Fig — (PDF) [file pone.0244005.s008.pdf]
