# Supplementary material for: DDRP: Real-time phenology and climatic suitability modeling of invasive insects
Source: PLoS One. 2020 Dec 31;15(12):e0244005. doi: 10.1371/journal.pone.0244005 (PMC7775054; doi:10.1371/journal.pone.0244005)
Supplement: S1 Table — (PDF) [file pone.0244005.s009.pdf]

S1 Table. Locality records used for validating the CLIMEX and DDRP climatic suitability models for *Neoleucinodes elegantalis.* The two-letter country code, locality name (if known), state or province, latitude, longitude, and reference for each record is indicated. The "Approx." column indicates whether geographic coordinates were approximated from specific location information such as a city name (Approx. = 1), or if they were reported by the study or database (Apprx. = 0).

Country	Locality	State/Province	Latitude	Longitude	Approx.	Reference
AR	Posadas	Misiones	-27.233	-55.567	0	1
AR	Orán	Salta	-22.871	-64.363	0	2
BR		Algoas	-10.371	-36.995	0	3
BR	Manaus	Amazonas	-3.120	-60.022	1	4
BR	Empresa Brasileira de Pesquisa Agropecuaria	Brasília	-15.462	-47.576	1	5
BR	Jardim Botanico de Brasília	Brasília	-15.862	-47.830	1	5
BR	Reserva Ecologica do IBGE	Brasília	-15.949	-47.878	1	5
BR	Camocim de São Félix	Ceará	-8.347	-35.774	1	6
BR	Croatá	Ceará	-4.400	-40.900	0	7
BR	Guaraciaba do Norte	Ceará	-4.170	-40.730	0	7
BR	Jaburuna, Ubajara	Ceará	-3.854	-40.921	0	8
BR	Tiangua	Ceará	-3.734	-40.996	1	9
BR	Alegre	Espírito Santo	-20.752	-41.489	0	10
BR	Domingos Martins	Espírito Santo	-20.364	-40.658	1	11
BR	Vargem Alta	Espírito Santo	-20.673	-41.010	1	11
BR	Fazenda Agua Limpa	Goiás	-15.330	-47.416	0	12
BR	Fazenda Andreia, Abadia de Goiás	Goiás	-16.836	-49.456	0	13
BR	Fazenda Experimental da empresa Unilever, Goiaia	Goiás	-16.718	-49.414	0	13
BR	Fazenda Santa Rosa das Flores, Palminopolis	Goiás	-16.766	-50.106	0	13
BR	Goianápolis	Goiás	-16.505	-49.021	1	14
BR	Universidade Federal de Goiás, Goiania	Goiás	-16.600	-49.278	1	15
BR	Dourados	Mato Grosso do Sul	-22.23	-54.802	1	16
BR	Coimbra	Minas Gerais	-20.857	-42.469	0	17
BR	Rio Paranaiba	Minas Gerais	-19.223	-46.205	0	18
BR	Vicosa	Minas Gerais	-20.813	-42.938	0	19
BR	Rodovia Transamazonica (Altamira/Itaituba)	Pará	-3.463	-52.895	1	20
BR	São José dos Pinhais	Paraná	-25.535	-49.206	0	21
BR	Encruzilhada de São João	Pernambuco	-8.244	-35.767	1	6

Country	Locality	State/Province	Latitude	Longitude	Approx.	Reference
BR	Garanhuns	Pernambuco	-8.883	-36.496	1	6
BR	Petrolina	Pernambuco	-9.383	-40.503	1	6
BR	Cha-Grande	Pernambuco	-8.233	-35.462	0	22
BR	Camocim de São Felix	Pernambuco	-8.359	-35.762	0	23
BR	Experimental Station of Vitória de Santo Antão	Pernambuco	-8.114	-35.291	0	24
BR	Serra Talhada	Pernambuco	-7.938	-38.313	0	25
BR	Centro de Pesquisa Mokiti Okada, Ipeúna	Rio de Janeiro	-22.402	-47.681	1	26
BR	Federal Rural University of Rio de Janeiro, Seropedica	Rio de Janeiro	-22.750	-43.683	0	27
BR	Itaperuna	Rio de Janeiro	-21.199	-41.892	1	28
BR		Rio de Janeiro	-22.451	-42.771	0	29
BR		Rio Grande do Sul	-28.054	-51.196	0	29
BR	University of Santa Maria campus Frederico Westphalen	Rio Grande do Sul	-27.397	-53.429	0	30
BR		Santa Catarina	-27.685	-48.497	0	29
BR	Experimental Station of Epagri, Lages	Santa Catarina	-27.800	-50.317	0	31
BR	São José do R. Pardo	São Paulo	-21.602	-46.904	1	6
BR	Divinolândia	São Paulo	-21.661	-46.735	1	32
BR	Taiúva	São Paulo	-21.124	-48.454	1	32
BR	Piracicaba	São Paulo	-22.823	-47.757	0	33
BR	Pongai	São Paulo	-21.733	-49.367	1	34
BR	Urupes	São Paulo	-21.202	-49.294	1	35
BR	Itabaiana	Sergipe	-10.689	-37.432	1	36
CO	El Peñol, La Piedra, El Porvenir	Antioquia	6.222	-75.179	0	2
CO	El Peñol, Santa Inés, La Gabriela	Antioquia	6.258	-75.273	0	2
CO	Jardín, La Linda, La Clara	Antioquia	5.614	-75.830	0	2
CO	El Penol, Santa Ines	Antioquia	6.257	-75.271	0	37
CO	Jardin, El Tapado/El Llano	Antioquia	5.601	-75.838	0	37
CO	Santo Domingo	Antioquia	6.477	-75.137	0	37
CO	Piedra Gorda	Antioquia	6.240	-75.480	1	38
CO	San Rafael	Antioquia	6.305	-75.025	0	39
CO	Buenavista, Patiño	Boyacá	5.496	-73.952	0	2
CO	Anserma, Palo Blanco Alto	Caldas	5.255	-75.769	0	2
CO	Anserma, San Pedro	Caldas	5.256	-75.797	0	2
CO	Manizales, El Rosario	Caldas	5.028	-75.583	0	2
CO	Manizales, San Peregrino	Caldas	5.057	-75.579	0	2
CO	Palestina, La Parroquia	Caldas	5.012	-75.631	0	2

Country	Locality	State/Province	Latitude	Longitude	Approx.	Reference
CO	Villa María, Tejares, Bachué	Caldas	5.032	-75.516	0	2
CO	Reserva Ecologico Rio Blanco	Caldas	5.126	-75.447	0	29
CO	Chinchina, San Andres/Los Alpes	Caldas	4.934	-75.614	0	37
CO	Manizales, Bajo Tablazo Hoyo Frio/Los Eucaliptos	Caldas	5.021	-75.533	0	37
CO	Inza, Belén	Cauca	2.473	-76.020	0	2
CO	Inza, Pedregal	Cauca	2.523	-76.000	0	2
CO	Nueva Estrella	Cordoba	9.288	-76.073	1	38
CO	Fusagasugá, Espinalito, La Poderosa	Cundinamarca	4.315	-74.417	0	2
CO	Fusagasugá, La Isla, El Recuerdo	Cundinamarca	4.349	-74.401	0	2
CO	San Bernardo, Pirineos bajos, Buenavista	Cundinamarca	4.151	-74.426	0	2
CO	Silvania, San Luis bajo	Cundinamarca	4.384	-74.630	0	2
CO	Silvania, San Luís bajo, Villa	Cundinamarca	4.704	-74.618	0	2
CO	Silvania, Santa Rita, Las Brisas	Cundinamarca	4.409	-74.361	0	2
CO	Silvania, Victoria Alta	Cundinamarca	4.424	-74.357	0	2
CO	Filandia, La Julia, Las Delicias	Departamento del Quindío	4.698	-75.682	0	2
CO	Pijao, La Playa, El Billar	Departamento del Quindío	4.335	-75.712	0	2
CO	Garzón, Fátima	Huila	2.181	-75.563	0	2
CO	Gigante, Bajo Corozal	Huila	2.339	-75.516	0	2
CO	Gigante, Tres Esquinas, La Ondina	Huila	2.310	-75.509	0	2
CO	Santa Marta, Minca, El Campano	Magdalena	11.124	-74.098	0	2
CO	Santa Marta, San Isidro, La Sirena	Magdalena	11.186	-74.009	0	2
CO	San Pedro de Cartago	Nariño	1.545	-77.116	0	2
CO	Arboleda	Nariño	5.583	-75.150	0	40
CO	La Florida	Nariño	1.302	-77.411	0	40
CO	La Union	Nariño	1.605	-77.134	0	40
CO	San Pedro de Cartago	Nariño	1.550	-77.119	0	40
CO	Tangua	Nariño	1.095	-77.394	0	40
CO	Yacuanquer	Nariño	1.115	-77.401	0	40
CO	Abrego, Llano Alto	Norte de Santander	8.077	-73.211	0	2
CO	Abrego, Los Piñitos	Norte de Santander	8.094	-73.238	0	2
CO	Ocaña, Llano Verde, Piedra gorda	Norte de Santander	8.405	-73.340	0	2
CO	Ocaña, Llano Verde, Piedra gorda	Norte de Santander	8.237	-73.357	0	2
CO	Pamplona, La Unión, La Morena	Norte de Santander	7.264	-72.577	0	2
CO	Silos, Cherqueta, La Vega	Norte de Santander	7.170	-72.742	0	2
CO	Dosquebradas, La Fría, Las Veraneras	Risaralda	4.854	-75.709	0	2

Country	Locality	State/Province	Latitude	Longitude	Approx.	Reference
CO	Dosquebradas, La Fría, Las Veraneras	Risaralda	4.855	-75.710	0	2
CO	Santa Rosa de Cabal, San Andrés, Los Alpes	Risaralda	4.935	-75.613	0	2
CO	Sta Rosa de Cabal/El Embol	Risaralda	4.920	-75.633	0	2
CO	Jesús María, Alto Grande, La Vega	Santander	5.879	-73.787	0	2
CO	Mesa de los Santos, Acuarela	Santander	6.759	-73.106	0	2
CO	Mesa de los Santos, Tabacal, El Alto	Santander	6.792	-73.056	0	2
CO	Pie de Cuesta, Sevilla	Santander	7.026	-72.994	0	2
CO	Cajamarca, Arenillal, La Esperanza	Tolima	4.391	-75.483	0	2
CO	Cajamarca, Arenillal, Mazatlán	Tolima	4.389	-75.484	0	2
CO	Cajamarca, La Esperanza	Tolima	4.440	-75.403	0	2
CO	Cajamarca, San Lorenzo Alto, Los Geranios	Tolima	4.447	-75.396	0	2
CO	Líbano, Santa Bárbara, Ambato	Tolima	4.944	-75.010	0	2
CO	Darién, La Playa	Valle del Cauca	3.959	-76.461	0	2
CO	Darién, La Unión, La Isabela	Valle del Cauca	3.956	-76.469	0	2
CO	Darién, La Unión, La Violeta	Valle del Cauca	3.925	-76.494	0	2
CO	La Unión, Córcega, Berlín	Valle del Cauca	4.534	-76.105	0	2
CO	Palmira, Corpoica	Valle del Cauca	3.517	-76.300	0	37
CR		Cartago	9.831	-83.563	0	29
CR		Cartago	9.784	-83.751	0	29
CU	Topes de Collantes	Sancti Spiritus	21.856	-79.990	0	41
EC	Caluma	Bolívar	-1.631	-79.258	1	42
EC	Chillanes	Bolívar	-1.944	-79.066	1	42
EC	Rioverde	Carchi	0.837	-78.372	0	2
EC		Carchi	0.840	-78.376	0	43
EC	La Maná	Cotopaxi	-0.941	-79.232	1	42
EC	Peñaherrera	Imbabura	0.350	-78.535	1	42
EC	Garcia Moreno	Imbabura	0.264	-78.646	1	42
EC	Palora	Morona Santiago	-1.699	-77.962	1	42
EC	Sucua	Morona Santiago	-2.456	-78.166	1	42
EC	Gualaquiza	Morona Santiago	-3.406	-78.572	1	42
EC		Morona Santiago	-1.460	-78.143	0	43
EC	AguaYaku, Salazar	Napo	-0.897	-77.770	0	2
EC	Cotundo, Aviles	Napo	-0.712	-77.585	0	2
EC	El Chaco, Llerena	Napo	-0.406	-77.838	0	2
EC	El Chaco, Sarrias	Napo	-0.359	-77.811	0	2

Country	Locality	State/Province	Latitude	Longitude	Approx.	Reference
EC	Guagua Sumaco	Napo	-0.736	-77.561	0	2
EC		Napo	-0.366	-77.817	0	43
EC		Napo	-0.904	-77.771	0	43
EC	Puyo	Pastaza	-1.494	-77.999	1	42
EC		Pastaza	-1.673	-77.960	0	43
EC	Pomona	Pastaza	-1.520	-77.371	0	44
EC	Los Bancos, Dr Chang	Pichincha	-0.013	-78.890	0	2
EC	Los Bancos, San Martín	Pichincha	-0.100	-79.013	0	2
EC	Tandapi	Pichincha	-0.414	-78.799	1	42
EC		Pichincha	-0.023	-78.894	0	43
EC	Saloya	Pichincha	-0.313	-78.710	0	44
EC	Cutuglagua, Mejia	Pichincha	-0.366	-78.550	0	45
EC	El Reventador	Sucumbíos	-0.035	-77.529	1	42
EC	Gonzalo Pizarro	Sucumbíos	0.016	-77.378	1	42
EC		Tungurahua	-1.426	-78.203	0	43
EC	Zamora	Zamora Chinchipe	-4.062	-78.950	1	42
GF			4.098	-52.680	0	29
GF			4.536	-52.123	0	29
GT		Jalapa	14.597	-89.983	0	29
GT	Suchitepe-quez, Santa Barbara Ref Quetzal UVG		14.541	-91.197	0	29
HN	Comayagua		14.460	-87.650	0	2
HO	Pico Bonito, Estación CURLA		15.697	-86.901	0	46
HO	Valle de Comayagua		14.336	-87.666	1	47
MX		Jalisco	20.337	-105.345	0	29
MX		Oaxaca	15.926	-96.418	0	29
MX		Veracruz	19.520	-96.942	0	29
MX	Misantla	Veracruz	19.925	-96.851	0	29
MX	Orizaba	Veracruz	18.848	-97.105	0	29
MX	Presidio	Veracruz	19.068	-96.973	0	29
PE	La Universidad Nacional Agraria de la Selva		-9.286	-75.998	0	48
PE	Nauto		-4.504	-73.583	1	49
PE	Loreto		-5.066	-73.851	1	49
PE	Tingo María, Huánuco		-9.896	-76.279	1	49
SR	Calcutta	Saramacca	5.833	-55.745	1	50
SR		Sipaliwini	5.067	-54.441	0	29

Country	Locality	State/Province	Latitude	Longitude	Approx.	Reference
TT	Curepe, Trinidad, W.I.		10.630	-61.400	0	29
TT	Morne Bleu Textel Installation, Trinidad		10.720	-61.290	0	29
TT	Palmiste, Trinidad, W.I.		10.240	-61.450	0	29
VE	Pao de Zarate	Aragua	10.114	-67.248	1	51
VE	Villa de Cura	Aragua	10.033	-67.493	1	52
VE	Valle del Turbio, Iribarren	Lara	10.066	-69.333	0	2
VE	El Tocuyo	Lara	9.783	-69.790	1	52
VE	El Cuji, Barquisimeto	Lara	10.156	-69.306	1	53
VE	El Molino, Jimenez	Lara	9.849	-69.613	1	54
VE	la Depresion de Quibor	Lara	9.933	-69.609	1	55
VE	San Juan de Lagunillas	Merida	8.499	-71.347	1	56
VE	El Corozo, Caripe	Monagas	9.694	-63.368	1	57
VE	Cordero	Táchira	7.855	-72.180	1	58
VE	Los Rios, Seboruco	Táchira	8.121	-72.157	0	59

References

1. Olckers T, Medal JC, Gandolfo DE. Insect herbivores associated with species of *Solanum* (Solanaceae) in Northeastern Argentina and southeastern Paraguay, with reference to biological control of weeds in South Africa and the United States of America. Florida Entomol. 2002;85: 254–260. Available from: https://journals.flvc.org/flaent/article/view/75070

2. Diaz-Montilla AE. Aspectas ecológicos y evolutivos del perforador del fruto *Neoleucinodes elegantalis* (Guenée) (Lepidoptera: Crambidae). PhD thesis, Universidad Nacional de Colombia, Bogotá, Colombia. 2016.

3. de Barros RP. Biologia da Broca Pequena (*Neoleucinodes elegantalis* Guenée, 1854): Estudo da Biologia da Broca Pequena (*Neoleucinodes elegantalis* Guenée, 1854) (Lepidoptera: Crambidae), em tomateiros. Novas Edições Acadêmicas; 2017.

4. Pamplona AMS, Berni RF, Chaves FCM, Oka JM. Pragas em pimentão sob cultivo protegido com uso deesterco e biofertilizante. Annals of LXI (61st) Annual Meeting of the Inter-American Society for Tropical Horticulture – ISTH. November 23-25, 2005; Brasília, Brazil, 2015. Available from: https://www.alice.cnptia.embrapa.br/bitstream/doc/1030253/1/AnaisISTHnov2015HT047.pdf (Accessed 2020 Sep 28)

5. Diniz IR, Morais HC, Camargo AJA. Host plants of lepidopteran caterpillars in the cerrado of the Distrito Federal, Brazil. Rev Bras Entomol. 2001;45: 107–122.

6. Maia AVP, Almeida C, Santoro KR, Melo JLA, Oliveira JV, Guedesd RNC, et al. High-level phylogeographic structuring of *Neoleucinodes elegantalis* Guenée (Lepidoptera, Crambridae) in Brazil: an important tomato pest. Rev Bras Entomol. 2016;60: 206–210. https://doi.org/10.1016/j.rbe.2016.03.004

7. Oliveira RCM, Pastori PL, Coutinho CR, Juvenal SO, Aguiar CVS. Natural parasitism of *Trichogramma pretiosum* (Hymenoptera: Trichogrammatidae) in *Neoleucinodes elegantalis* (Lepidoptera: Crambidae) eggs on tomato (Solanales: Solanaceae) in the Northeast region, Brazil. Braz. J. Biol. 2020;80: 474–475. https://doi.org/10.1590/1519-6984.206676

8. Filgueiras RMC. Tecido-não-tecido (TNT) para ensacamento de cachos visando controle de broqueadores de frutos do tomateiro (*Solanum lycopersicum* L.). PhD thesis, Universidade Federal do Ceará Centro, Fortaleza, Brazil. 2016. Available from: http://repositorio.ufc.br/handle/riufc/16449

9. Carneiro JS, Haji FNP, Santos FAM. Bioecologia e controle da broca pequena *Neoleucinodes elegantalis.* Teresina: Embrapa Meio Norte. Circular Técnica. 1998;26: 1–14. Available from: http://www.infoteca.cnptia.embrapa.br/infoteca/handle/doc/63866

10. Pratissoli D, Kloss TG, Zinger FD, Vianna UR. Does mating interfere in the biological characteristics of a population of *Trichogramma pretiosum*? 2014;86: 459–464. https://doi.org/10.1590/0001-3765201496712

11. Fragoso DFM, Pratissoli D, Araujo LM De, Damascena AP, Carvalho JR De, Zago HB. Thermal requirements, life table and estimate of number of *Trichogramma galloi* in eggs of *Neoleucinodes elegantalis*. Int J Adv Eng Res Sci. 2019;6495: 374–379. https://dx.doi.org/10.22161/ijaers.612.39

12. Diniz I, Morais H. Local pattern of host plant utilization by lepidopteran larvae in the cerrado vegetation. Entomotrópica Rev Int para el Estud la Entomol Trop. 2002;17: 115–119. Available from: https://tspace.library.utoronto.ca/handle/1807/5765

13. Camargo, AC. Monitoramento e manejo de insetos pragas em cultivos de tomate para processamento industrial. PhD thesis, Universidade Federal de Goiás, Goiânia, Goiás, Brazil. 2011. Available from: https://repositorio.bc.ufg.br/tede/handle/tede/6790

14. Santos MM, Noronha JF. Diagnóstico da cultura do tomate de mesa no município de Goianápolis, estado de Goiás, Brasil. Pesqui Agropecuária Trop. 2001;31: 29–34. Available from: https://www.revistas.ufg.br/index.php/pat/article/view/2523

15. Júnior PS, da Silva AL, Alcântara VED, Eduardo V, de Farias TA. Ensaio para o controle químico da broca pequena *Neoleucinodes elegantalis* (Guennée, 1854) – Pyralidae-Lepidoptera) do tomate. Pesqui Agropecu Trop. 1991-92;21/22: 127–131. Available from: https://www.revistas.ufg.br/pat/article/view/2609

16. Felix CDS, Pereira VL, de Aquino S, Cardoso ML, Uchôa-Fernandes MA. Moscas colonizando frutos nativos e cultivados na região de dourados, Mato Grosso do Sul. VI Congresso de Ecologia do Brasil, Fortaleza, Brazil (Volume 1). 2003; 415–416. Available from: http://seb-ecologia.org.br/revistas/indexar/anais/2003/3.pdf (Accessed 2020 Sep 28)

17. Silva ÉM, Silva RS, Gontijo PC, Rosado JF, Bacci L, Martins JC, et al. Climatic variables limit population abundance of *Neoleucinodes elegantalis*: Important neotropical tomato pest. Crop Prot. 2020;138: 105325. https://doi.org/10.1016/j.cropro.2020.105325

18. Lima ELF. Distribuição espacial e unidade amostral de Lepidópteros broqueadores em tomateiro (*Solanum lycopersicum*). MS thesis, Universidade Federal de Viçosa, Rio Paranaíba. 2019. Available from: https://www.locus.ufv.br/bitstream/handle/123456789/25642/texto%20completo.pdf?sequence=1

19. Ramos RS, Picanço MC, Santana PA, Júnior ÉMS, Bacci L, Rocha AHG, et al. Natural biological control of lepidopteran pests by ants. Sociobiology. 2012;59: 1389–1399. Available from: http://periodicos.uefs.br/ojs/index.php/sociobiology/article/view/511/453

20. Kato M do SA, Poltronieri MC. Pragás do tomateiro em Altamira, Pará. Simpósio do Trópico Úmido, Anais Proceedings Anales Volume III, Culturas Temporárias. Brasília, DF; 1984. November 12−17, 1984; Brasília, Brazil. Available from: https://www.alice.cnptia.embrapa.br/bitstream/doc/394636/1/CPATUDoc36v3P293.pdf (Accessed 2020 Sep 28)

21. Moraes CP. Flutuação populacional e biologia de *Neoleucinodes elegantalis* (Guenée) (Lepidoptera: Crambidae) em cultivo de tomate (*Solanum lycopersicum* L.). Universidade Federal do Parana. PhD thesis, Universidade Federal do Paraná, Curitiba, Brazil. 2014. Available from: https://www.acervodigital.ufpr.br/handle/1884/35758

22. Correia AMO. Biologia e técnica de criação de *Neoleucinodes elegantalis* (Guenée) em hospedeiros naturais. MS thesis, Universidade Federal Rural de Pernambuco, Recife, Brazil. 2013. Available from: http://www.ppgea.ufrpe.br/sites/ppgea.ufrpe.br/files/documentos/auridete.pdf (Accessed 2020 Sep 28)

23. Alves SM. Manejo de *Neoleucinodes elegantalis* (Guenée) (Lepidoptera: Crambidae) no agreste de Pernambuco com *Trichogramma pretiosum* Riley (Hymenoptera: Trichogrammatidae) e produtos fitossanitários seletivos. PhD thesis, Universidade Federal Rural de Pernambuco, Recife, Brazil. 2016. Available from: http://ppgea.ufrpe.br/sites/ppgea.ufrpe.br/files/documentos/solange_maria_de_franca_0.pdf (Accessed 2020 Sep 28)

24. de Lyra Netto AMC, Lima AAF. Infestação de cultivares de tomateiro por *Neoleucinodes elegantalis* (Lepidoptera: Pyralidae). Pesqui Agropecu Bras. 1990;33: 221–223. Available from: https://seer.sct.embrapa.br/index.php/pab/article/view/4840

25. Anselmo WM. Efeito de extratos vegetais de flor de seda e juazeiro nomanejo de pragas na cultura do tomateiro. MS thesis, Universidade Federal Rural de Pernambuco, Serra Talhada. 2013. Available from: http://www.tede2.ufrpe.br:8080/tede2/handle/tede2/6173

26. Gomes FB, Fortunato LJ, Pacheco ALV, Azevedo LH, Freitas N, Homma SK. Incidência de pragas e desempenho produtivo de tomateiro orgânico em monocultivo e policultivo. Hortic Bras. 2012;30: 756–761. https://doi.org/10.1590/S0102-05362012000400032

27. Genuncio GC, Silva ES, Nascimento EC, Zonta E, Araújo AP. Spring-summer tomato yield as a function of potassium fertilization in field and protected crops. African J Agric Res. 2014;9: 2511–2119. https://doi.org/10.5897/AJAR2013.7862

28. Eiras ÁE. Calling behaviour and evaluation of sex pheromone glands extract of *Neoleucinodes elegantalis* Guenée (Lepidoptera: Crambidae) in wind tunnel. An da Soc Entomológica do Bras. 2000;29: 453–460. https://doi.org/10.1590/S0301-80592000000300007

29. GBIF.org (19 August 2020) GBIF Occurrence Download https://doi.org/10.15468/dl.uq7ey2

30. Schmidt D, Zamban DT, Prochnow D, Caron BO, Souza VQ, Paula GM, et al. Caracterização fenológica, filocrono e requerimento térmico de tomateiro italiano em dois ciclos de cultivo. Hortic Bras. 2017;35: 89–96. https://doi.org/10.1590/s0102-053620170114

31. Modolon TA, Boff P, Boff MI, Miquelluti DJ. Homeopathic and high dilution preparations for pest management to tomato crop under organic production system. Hortic Bras. 2012;1: 51–57. https://doi.org/10.1590/S0102-05362012000100009

32. Medal JC, Charudattan R, Mullahey JJ, Pitelli RA. An exploratory insect survey of tropical soda apple in Brazil and Paraguay. Florida Entomol. 1996;79: 70–73. https://doi.org/10.2307/3495757

33. Araujo JC, Telhado SFP, Sakai RH, Ledo CAS, Melo PCT. Univariate and multivariate procedures for agronomic evaluation of organically grown tomato cultivars. Comun científica. 2016;34: 374–80. https://doi.org/10.1590/S0102-05362016003011

34. Júnior PS, da Silva AL, Alcântara VED, de Farias TA. Ensaio para o controle químico da broca pequena *Neoleucinodes elegantalis* (Guennée 1854) (Pyralidae-Lepidoptera) do tomate. Pesqui Agropecuária Trop. 2007;21: 127–131. Available from: https://www.revistas.ufg.br/pat/article/view/2609

35. de Lima MF, Boiça Jr AL, de Souza RS. Efeito de inseticidas no controle da broca pequena Neoleucinodes elegantalis na cultura do tomateir. Rev Ecossistema. 2001;26: 54–57.

36. Nunes MUC, Leal MLS. Efeito da aplicação de biofertilizante e outros produtos químicos e biológicos, no controle da broca pequena do fruto e na produção do tomateiro tutorado em duas épocas de cultivo e dois sistemas de irrigação. Hortic Bras. 2001;19: 53–59. https://doi.org/10.1590/S0102-05362001000100011

37. Díaz-Montilla AE, Baena-Bejarano N, Montoya-Lerma J, Saldamando-Benjumea CI. Reproductive incompatibility and fitness components in *Neoleucinodes elegantalis* races (Lepidoptera, Crambidae) from three solanaceae hosts. Caldasia. 2018;40: 199–215. http://dx.doi.org/10.15446/caldasia.v40n2.67241

38. Díaz AEM, Solis A, Brochero HL. Distribución geográfica de *Neoleucinodes elegantalis* (Lepidoptera: Crambidae) en Colombia. Rev Colomb Entomol. 2011;37: 71–76. Available from: http://www.scielo.org.co/scielo.php?script=sci_arttext&pid=S0120-04882011000100012&lng=en&nrm=iso

39. Perea Valois AP, Mosquera CAM, Ocampo MLO. Evaluación del extracto de anamú (*Petiveria alliacea*), en el control del gusanoperforador (*Neoleucinodes elegantalis*) en el cultivo de tomate (*Solanum lycopersicum*). MS thesis, Universidad Pontificia Bolivariana, Medellín, Colombia. 2017. Available from: https://repository.upb.edu.co/handle/20.500.11912/3400

40. Santander LKL, Cabrera FAV, Burbano TCL, Alvarado DED. Evaluación agronómica de familias de medios hermanos de lulo de Castilla, *Solanum quitoense* Lam. Rev UDCA Actual Divulg Científica. 2020;23: e1334. https://doi.org/10.31910/rudca.v23.n1.2020.1334

41. Águila RN. Lepidoptera (Insecta) de Topes de Collantes, Sancti Spiritus, Cuba. Boletín la SEA. 2004;34: 151–159. Available from: http://sea-entomologia.org/PDF/BOLETIN_34/B34-025-151.pdf (Accessed 2020 Sep 28)

42. Basantes MAN. Caracterización morfométrica de la biodiversidad del barrenador del fruto *Neoleucinodes elegantalis* (Guneeé) en el cultivo de naranjilla *Solanum quitoense* en diez localidades. MS thesis, Universidad Central del Ecuador, Quito, Ecuador. 2015. Available from: http://www.dspace.uce.edu.ec/handle/25000/4794

43. Noboa M, Díaz A, Vásquez W, Viera W. Parasitoids of *Neoleucinodes elegantalis* Guneeé (Lepidoptera: Crambidae) in Ecuador. Idesia (Arica). 2017;35: 49–54. http://dx.doi.org/10.4067/S0718-34292017005000015

44. Larrea MBL. Evaluación de la eficiencia de extractos vegetales y agentes microbiológicos para el control del barrenador del fruto de la Naranjilla *Neoleucinodes elegantalis.* MS thesis, Universidad Central del Ecuador, Quito, Ecuador. 2015. Available from: http://www.dspace.uce.edu.ec/handle/25000/4685

45. Segovia SGY. Evaluación de un virus entomopatógeno como potencial agente de control biológico de *Neoleucinodes elegantalis* (Guenée) (Lepidoptera: Crambidae). MS thesis, Universidad Austral de Chile, Valdivia, Chile. 2012. Available from: http://cybertesis.uach.cl/tesis/uach/2012/egy.24e/doc/egy.24e.pdf (Accessed 2020 Sep 28)

46. Miller JY, Matthews DL, Warren, AD, Solis A, Harvey DJ, Gentili-Poole P, et al. An annotated list of the Lepidoptera of Honduras. Insecta Mundi. Paper 725. 2012. Available from: http://digitalcommons.unl.edu/insectamundi/725

47. Espinoza HR. Barrenador del fruto de la berenjena, *Neoleucinodes elegantalis*. Fundación Hondureña de Investigación Agrícola (FHIA), La Lima, Cortés, Honduras. Hoja Técnica No. 2. 2008. Available from: http://v2.fhia.info/dowloads/proteccion_veg_pdfs/Hojaprotvegetal2.pdf (Accessed 2020 Sep 28)

48. Anteparra M, Ruiz S, Diaz W. Entomofauna asociada con la cocona (*Solanum sessiliflorum* Dunal) en Tingo María, Huánuco. Investig Amaz. 2012;2: 51–59. Available from: http://localhost:8080/xmlui/handle/123456789/512

49. Delgado-Vásquez C, Couturrier G, Anteparra M. Principales fitófagos de la cocona *Solanum sessiliforum dunal* (Solanaceae) en la Amazonía Peruana. Folia Amaz. 2011;20: 45–51. https://doi.org/10.24841/fa.v20i1-2.316

50. de Putter H, Wongsonadi H. Invloed van ras, mulch en bemesting op de productie en het saldo van tomaat: verslag van een proef te Saramacca, Suriname. Nederland: Praktijk Plant & Omgeving. Akkerbouw, Groene ruimte en Vollegrondsgroenten. 2009. Available from: https://edepot.wur.nl/135548

51. Jaffe K, Mirás B, Cabrera A. Mate selection in the moth *Neoleucinodes elegantalis*: evidence for a supernormal chemical stimulus in sexual attraction. Anim Behav. 2007;73: 727–734. https://doi.org/10.1016/j.anbehav.2006.10.011

52. Cermeli M, Ramirez E, Van Balen L, Geraud F, Garcia D, Sandoval JR. Problemas encontrados en el control químico de plagas de tomate en dos regiones de Venezuela. Ciarco. 1972;2: 76–84.

53. Fernández S, Salas JA. Estudios sobre la biologia del perforador del fruto del tomate *Neoleucinodes elegantalis* Guenée (Lepidoptera: Pyraustidae). Agron Trop. 1985;35: 77–82.

54. Paz R, Arrieche, N. Presencia y daño de *Neoleucinodes elegantalis* (Lepidoptera: Crambidae) en un sistema de producción de tomate bajo condiciones de umbráculo cerrado. Resúmenes del XIX Congreso Venezolano de Entomología. July 4–7, 2005; San Felipe Estado Yaracuy, Venezuela. Available from: http://www.bioline.org.br/pdf?em05022

55. Fernández S, Salas J, Álvarez C, Parra A. Fluctuación poblacional de los principales insectos-plagas del tomate en la Depresión de Quíbor, Estado Lara, Venezuela. Agron Trop. 1987;37: 31–42.

56. Brienco Vergara AJ, Suarez F. Insectos comunes del tomate cultivado en San Juan de Lagunillas, estado Merida. Jornadas Agronomicas. October 12–15, 1977; Maracay, Venezuela.

57. de la Lama, KAA, Vásquez GLN. Niveles de infestación de *Neoleucinodes elegantalis* Geneé en frutos de lulo Solanum quitoense Lam var. Quitoense y var. Septentrionale en el municipio Caripe del estado Monagas. Resúmenes del XIX Congreso Venezolano de Entomología. July 4–7, 2005; San Felipe Estado Yaracuy, Venezuela. Available from: http://www.bioline.org.br/pdf?em05022

58. Sánchez J, Pallares J. Evaluación de métodos para el control de *Neoleucinodes elegantalis* en el cultivo de tomate, de la finca "La Primavera", Cordero, estado Táchira. Resúmenes del XIX Congreso Venezolano de Entomología. July 4–7, 2005; San Felipe Estado Yaracuy, Venezuela. Available from: http://www.bioline.org.br/pdf?em05022

59. Alarcón IS. Evaluación del control biológico, etológico y químico de *Neoleucinodes elegantalis* en tomate (*Lycopersicon esculentum*). Rev Ambient. 2020;3: 31–40. Available from: http://unellez.edu.ve/revistas/index.php/ambientellania/article/view/888
