# Supplementary material for: DDRP: Real-time phenology and climatic suitability modeling of invasive insects
Source: PLoS One. 2020 Dec 31;15(12):e0244005. doi: 10.1371/journal.pone.0244005 (PMC7775054; doi:10.1371/journal.pone.0244005)
Supplement: S2 Table — (PDF) [file pone.0244005.s010.pdf]

S2 Table. **Comparison of DDRP predictions for the dates of first spring egg laying ("Predicted") to the month (data set 1) or dates (data sets 2 and 3) of peak spring adult flight for *Epiphyas postvittana* in California according to three monitoring data sets ("Observed").** For monitoring data sets 1 and 2, DDRP predictions for each region are averages (and range) of several grid cells (see S2 Appendix). For data sets 2 and 3, the difference in days ("Diff") between DDRP predictions and dates of peak spring flight are presented.

Year	Data set	Region	Observed	Predicted	Min	Max	Diff	Timing
2008	1	Alameda Co.	May	4/01	3/27	4/07		Early
		Contra Costa Co.	May	4/15	3/27	5/19		In range
		Monterey Co.	Apr	4/12	3/17	5/13		In range
		San Francisco Co.	May	4/12	3/30	5/14		In range
2009	1	Alameda Co.	Apr	3/21	3/19	3/26		Early
		Contra Costa Co.	May	4/06	3/18	5/10		In range
		Monterey Co.	May	4/02	3/02	5/05		In range
		San Francisco Co.	Apr	4/07	3/21	5/10		In range
2012	2	Region 2	3/01	3/23	3/19	4/17	22	
		Region 3	3/01	3/23	3/17	4/10	22	
		Region 4	3/01	3/22	3/21	3/24	21	
		Region 5	3/01	3/26	3/21	3/30	25	
						Average	22.5	Late
2013	2	Region 1	4/10	4/10	3/27	4/25	0	
		Region 2	4/10	3/29	3/27	4/08	−12	
		Region 3	4/10	4/05	3/27	4/25	−5	
		Region 4	4/10	3/31	3/30	4/01	−10	
		Region 5	4/10	4/09	3/31	4/14	−1	
						Average	−5.6	Early
2014	2	Region 3	3/03	3/06	3/01	3/17	3	
		Region 5	3/03	3/07	3/02	3/09	4	
						Average	3.5	Late
2019	3	Salinas	3/28	3/23			−5	Early
2020	3	Salinas	3/13	3/15			2	Late
