# Supplementary material for: DDRP: Real-time phenology and climatic suitability modeling of invasive insects
Source: PLoS One. 2020 Dec 31;15(12):e0244005. doi: 10.1371/journal.pone.0244005 (PMC7775054; doi:10.1371/journal.pone.0244005)
Supplement: S3 Table — (PDF) [file pone.0244005.s011.pdf]

S3 Table. DDRP predictions of the number of degree-days Celsius (DDC) that accumulated between peaks in last fall flight and first spring flight of *Epiphyas postvittana* in California. For monitoring data set 2, predictions for each region are averages of four DDRP grid cells (see S2 Appendix). Missing results for some regions are due to indiscernible peaks in flight for one or both seasons.

Years	Data set	Region	Fall peak	Spring peak		DDC
2011–2012	2	Region 2	10/13	3/01		695
		Region 3	10/13	3/01		689
		Region 4	10/13	3/01		705
		Region 5	10/13	3/01		684
					Average	693
2012–2013	2	Region 1	10/06	4/10		848
		Region 2	11/06	4/10		688
		Region 3	10/21	4/10		758
		Region 4	10/06	4/10		977
		Region 5	10/06	4/10		866
					Average	827
2013–2014	2	Region 3	10/13	3/03		759
		Region 5	10/13	3/03		728
					Average	744
2019–2020	3	Salinas	10/18	3/15		823
