# Supplementary material for: DDRP: Real-time phenology and climatic suitability modeling of invasive insects
Source: PLoS One. 2020 Dec 31;15(12):e0244005. doi: 10.1371/journal.pone.0244005 (PMC7775054; doi:10.1371/journal.pone.0244005)
Supplement: Attachment — Submitted filename: Response_to_Reviewers.pdf [file pone.0244005.s012.pdf]

PONE-D-20-15142

DDRP: real-time phenology and climatic suitability modeling of invasive insects

PLOS ONE

Dear Dr. Barker,

Thank you for submitting your manuscript to PLOS ONE. After careful consideration, we feel that it has merit but does not fully meet PLOS ONE's publication criteria as it currently stands. Therefore, we invite you to submit a revised version of the manuscript that addresses the points raised during the review process.

The reviewers and I agree that this a well-written manuscript. I appreciate your attention to detail and your thorough explanations of the choices you made in developing DDRP. However, Reviewer 1 expressed reservations about the lack of underlying hypotheses and validation, and I share some of those concerns. As you are probably aware, PLOS ONE has a specific set of criteria for manuscripts that present new software. These criteria relate to utility, validation, and availability (https://journals.plos.org/plosone/s/submission-guidelines#loc-methods-software-databases-and-tools). With respect to validation, "the submitted manuscript must demonstrate that the new tool is an improvement over existing options in some way. This requirement may be met by including a proof-of-principle experiment or analysis; if this is not possible, a discussion of the possible applications and some preliminary analysis may be sufficient." I believe your goal is to see wide uptake of DDRP, which means that you must meet a fairly high standard for validation that goes beyond preliminary analysis. Certainly, your discussion of the practical advantages of DDRP over alternatives is compelling, but it would be much better if you provided quantitative evidence.

I encourage you to look to the comments of Reviewer 1 for potential opportunities to evaluate model performance. For instance, it should be relatively straightforward to implement (holdout) validation of the predicted distributions for your two example cases and derive some estimates of the error rates. Additionally, you should be able to devise some sort of test of the relative merit of your degree-day modeling method, even with limited data.

Please submit your revised manuscript by Aug 31 2020 11:59PM. If you will need more time than this to complete your revisions, please reply to this message or contact the journal office at plosone@plos.org. When you're ready to submit your revision, log on to https://www.editorialmanager.com/pone/ and select the 'Submissions Needing Revision' folder to locate your manuscript file.

Please include the following items when submitting your revised manuscript:

- A rebuttal letter that responds to each point raised by the academic editor and reviewer(s). You should upload this letter as a separate file labeled 'Response to Reviewers'.
- A marked-up copy of your manuscript that highlights changes made to the original version. You should upload this as a separate file labeled 'Revised Manuscript with Track Changes'.

- An unmarked version of your revised paper without tracked changes. You should upload this as a separate file labeled 'Manuscript'.

If you would like to make changes to your financial disclosure, please include your updated statement in your cover letter. Guidelines for resubmitting your figure files are available below the reviewer comments at the end of this letter.

If applicable, we recommend that you deposit your laboratory protocols in protocols.io to enhance the reproducibility of your results. Protocols.io assigns your protocol its own identifier (DOI) so that it can be cited independently in the future. For instructions see: http://journals.plos.org/plosone/s/submission-guidelines#loc-laboratory-protocols

We look forward to receiving your revised manuscript.

Kind regards,

Frank H. Koch, PhD

Academic Editor

PLOS ONE

RESPONSE TO EDITOR

The reviewers and I agree that this a well-written manuscript. I appreciate your attention to detail and your thorough explanations of the choices you made in developing DDRP. However, Reviewer 1 expressed reservations about the lack of underlying hypotheses and validation, and I share some of those concerns. As you are probably aware, PLOS ONE has a specific set of criteria for manuscripts that present new software. These criteria relate to utility, validation, and availability (https://journals.plos.org/plosone/s/submission-guidelines#loc-methods-software-databases-and-tools). With respect to validation, "the submitted manuscript must demonstrate that the new tool is an improvement over existing options in some way. This requirement may be met by including a proof-of-principle experiment or analysis; if this is not possible, a discussion of the possible applications and some preliminary analysis may be sufficient." I believe your goal is to see wide uptake of DDRP, which means that you must meet a fairly high standard for validation that goes beyond preliminary analysis. Certainly, your discussion of the practical advantages of DDRP over alternatives is compelling, but it would be much better if you provided quantitative evidence.

- **Response:** We are grateful for the editor's and reviewers' insightful suggestions. Our revised manuscript now meets the specific set of criteria for manuscripts that present new software:

  1. **Utility:** We have now included several model validation analyses in our study, which show that DDRP can correctly predict the distribution and phenology of the focal species. Additionally, we have expanded our discussion on the advantages of DDRP over other currently available platforms in a new section of the Discussion ("Comparison of DDRP to other platforms"). Importantly, there are no existing alternatives to DDRP, which we have more clearly emphasized in our revised manuscript. Additionally, we are unaware of any publicly available models for the two focal species (*E. postvittana* and *N. elegantalis*) that generate predictions that are comparable to DDRP, so we cannot test whether the DDRP method is better, worse, or the same as other methods.

  2. **Validation:** As described in more detail in our responses to Reviewer 1, we have set up and tested hypotheses related to the ability of DDRP to:
     a. Correctly predict the known distribution of each focal species using a set of validation localities from areas that were not used for model development. For small tomato borer (*N. elegantalis*), which is not (yet) present in CONUS, we validated a climatic suitability model for the species in Brazil, which also highlights DDRP's ability to be used for regions outside of CONUS. DDRP predictions of the potential distribution of both species for multiple recent years were in agreement with the vast majority of validation localities. This finding supports our hypothesis that DDRP can correctly predict the known distribution of each species.

        Moreover, we calibrated DDRP climatic suitability models in accordance with improved CLIMEX models for each species, and found that DDRP can predict a similar potential distribution as CLIMEX (using 30-year climate normals centered on 1975). This finding provides another source of validation that DDRP is capable of modeling climatic suitability.

b. Correctly predict the timing of first spring egg laying and the generation length of *E. postvittana*. This hypothesis was generally supported, although there was variation in prediction error across years and regions for multiple potential reasons (see 'Discussion'). Unfortunately, we were unable to find monitoring data that were suitable for validating the DDRP phenology model for *N. elegantalis.*

3. **Availability:** DDRP is written in R, which is a widely used and popular (in Universities and the scientific community) open source statistical and programming language. The most current version is available at GitHub and the version used for this study have been archived at Zenodo. The 30-year average PRISM data and associated scripts used to produce these data are also available at these repositories.

I encourage you to look to the comments of Reviewer 1 for potential opportunities to evaluate model performance. For instance, it should be relatively straightforward to implement (holdout) validation of the predicted distributions for your two example cases and derive some estimates of the error rates. Additionally, you should be able to devise some sort of test of the relative merit of your degree-day modeling method, even with limited data.

- **Response:** As noted in our previous response and in our response to Reviewer 1, we have now validated the DDRP climatic suitability models for both focal species (which include estimates of error), and we validated predictions of the DDRP phenology model for *E. postvittana*.

2. We note that [Figure(s) S1, S2, S4, 2, 3, 4 and 5] in your submission contain [map/satellite] images which may be copyrighted. All PLOS content is published under the Creative Commons Attribution License (CC BY 4.0), which means that the manuscript, images, and Supporting Information files will be freely available online, and any third party is permitted to access, download, copy, distribute, and use these materials in any way, even commercially, with proper attribution. For these reasons, we cannot publish previously copyrighted maps or satellite images created using proprietary data, such as Google software (Google Maps, Street View, and Earth). For more information, see our copyright guidelines: http://journals.plos.org/plosone/s/licenses-and-copyright.

- **Response:** We do not need to request copyright permission for any figures in this manuscript because they have not been published previously. Several parameter values used for our CLIMEX modeling analyses were derived from earlier studies, which is perhaps why there was confusion about this. We have edited the captions of figures that present CLIMEX maps to clarify this point.

Additional Editor Comments (if provided):

Minor comments:

Table 1 – references/citations for these parameter values? (I realize they're provided later but it would be good to include references in the caption for readers)

- **Response:** We made this correction.

Line 215 – delete “estimates of” (redundant)

- **<u>Response</u>:** We made this correction.

Line 271- “high” instead of “highly”

- **<u>Response</u>:** We made this correction.

[Note: HTML markup is below. Please do not edit.]

Reviewers' comments:

Reviewer's Responses to Questions

Comments to the Author

1. Is the manuscript technically sound, and do the data support the conclusions?

The manuscript must describe a technically sound piece of scientific research with data that supports the conclusions. Experiments must have been conducted rigorously, with appropriate controls, replication, and sample sizes. The conclusions must be drawn appropriately based on the data presented.

Reviewer #1: No

Reviewer #2: Yes

---

2. Has the statistical analysis been performed appropriately and rigorously?

Reviewer #1: No

Reviewer #2: N/A

---

3. Have the authors made all data underlying the findings in their manuscript fully available?

The PLOS Data policy requires authors to make all data underlying the findings described in their manuscript fully available without restriction, with rare exception (please refer to the Data Availability Statement in the manuscript PDF file). The data should be provided as part of the manuscript or its supporting information, or deposited to a public repository. For example, in addition to summary statistics, the data points behind means, medians and variance measures should be available. If there are restrictions on publicly sharing data—e.g. participant privacy or use of data from a third party—those must be specified.

Reviewer #1: Yes

Reviewer #2: Yes

---

4. Is the manuscript presented in an intelligible fashion and written in standard English?

PLOS ONE does not copyedit accepted manuscripts, so the language in submitted articles must be clear, correct, and unambiguous. Any typographical or grammatical errors should be corrected at revision, so please note any specific errors here.

Reviewer #1: Yes

Reviewer #2: Yes

---

5. Review Comments to the Author

Please use the space provided to explain your answers to the questions above. You may also include additional comments for the author, including concerns about dual publication, research ethics, or publication ethics. (Please upload your review as an attachment if it exceeds 20,000 characters)

RESPONSE TO REVIEWER 1

Reviewer #1: Barker et al. provide a well written accounting of DDRP, a new software package to calculate degree days and climatic suitability for insects. The authors are correct that refinements are needed to prepare forecasts of the potential distribution and activity of insects over time. Such forecasts could be useful to refine surveys, particularly for invasive species whose complete current distribution may not be known with confidence.

While I do not find any errors, per se, I do not find any underlying hypotheses to underpin the research. One set of hypotheses could be related to the ability of the model to reproduce the distribution or activity of the light brown apple moth or the small tomato borer, the example cases that were provided. Ideally, these forecasts would be for areas that were not used for model development. Such validation is routine for these types of models and is a bare minimum. The model might have high commission error rates, classifying more areas as suitable than exist. It is not clear how well the model performs with respect to seasonal activity/phenology.

- **Response:** Our work on DDRP has been primarily supported by the CAPS program at USDA APHIS PPQ, so it was designed for species (15 to date) that are not (yet) established in CONUS, with the exception of light brown apple moth (*E. postvittana*). Validating a model for a not-yet present species such as the small tomato borer (*N. elegantalis*) for CONUS is therefore impossible. However, we strongly agree with the reviewer that model validation is very important, and we have now included the following three DDRP model validation analyses in our manuscript:

    1) We contacted several people to obtain trapping data for *E. postvittana* and were able to acquire three data sets from an individual at APHIS. We used information on peak catches over a 12 year time frame (2008–2009, 2011–2014, and 2019–2020) to validate DDRP predictions of first spring egg laying and generation time (see S2 Appendix). Please note that we have not yet been given guidance as to whether we can share these government collected data (mostly Excel plots).
    2) We validated the DDRP climatic suitability model for *E. postvittana* using hundreds of presence locations from California.
    3) We validated the DDRP climatic suitability model for *N. elegantalis* by modeling the species in Brazil and assessing whether predictions were consistent with known presence locations, general knowledge on the distribution of the species in this country, and predictions of suitability according to CLIMEX.

    Unfortunately, we were unable to find monitoring data that were suitable for validating the DDRP phenology model for *N. elegantalis*. Population monitoring studies of the species have been conducted in Brazil; however, the species at these locations does not have a discrete overwintering stage so peaks in flight are more or less sporadic and not indicative of first spring activity or voltinism. We emphasize, however, that the phenology model uses published data from multiple sources to derive parameters, and that our presentation of the model via a publication will provide a first draft that can later be validated and further calibrated if and when the species arrives in CONUS. This of course is true for all DDRP models that are presented in this study and at the homepage for DDRP (http://uspest.org/CAPS/).

Moreover, we have conducted additional calibration and validation analyses to the CLIMEX models for both focal species. The ability of DDRP to predict a very similar potential distribution as the validated CLIMEX model for CONUS is in itself a source of validation that the platform can accurately predict climatic suitability, which we have stated in the revised manuscript.

With respect to the comment about setting up hypotheses, we have now included hypotheses in the last paragraph of the Introduction. However, we'd also like to point out that models themselves can serve as hypotheses and toolkits that can then be used to set up studies using more specific hypotheses. Simulation modeling changes the way that problems are conceived and researched: we can formulate and understand a problem by modeling it, and then challenge the model with new data, rather than by using a more traditional mode of starting with observations and following up with hypotheses (see Hilborn and Mangel, 1997, The Ecological Detective: Confronting Models with Data (MPB-28). Princeton University Press, Princeton, NK).

The authors discuss at great length different methods of estimating degree days. An argument is made that the method of calculation could result in quantitatively and qualitatively different forecasts. Such a suggestion is intriguing (and is certainly the subject of several other research papers), but the authors provide no new evidence (e.g., trap catch data over time) to demonstrate that the method they selected was substantially better, worse, or no different from other methods.

- **Response:** We are not completely clear on which exact sentences or section of the manuscript this comment is referring to, but perhaps it is referring to methods to calculate degree-days. As described in the Methods, DDRP offers three calculation methods: simple average, the simple average using an upper threshold with a horizontal cutoff, and the single triangle method with upper threshold. Users may also add their own calculation method if they wish. We used the single triangle method for our focal species models, which we have now clarified in the revision. This method has high performance (e.g., Cesaraccio et al. 2001. Int J Biometeorol 45:161-169) and is a recommended standard for IPM modeling tools. However, a comparison of how predictions for the two focal species may vary when we calculate degree-days using different methods is beyond the scope of this study.

  If the comment is referring to why we did not compare model predictions of DDRP to those of other platforms, then we want to emphasize that 1) there are no platforms with DDRP's capabilities (i.e. that can generate and integrate phenology and climate suitability models in real-time), and 2) there are no available degree-day models for our focal species. We therefore cannot test whether the DDRP method is better, worse, or the same as other methods. We have expanded our discussion of how DDRP compares to other currently available platforms in the Discussion (see "Comparison of DDRP to other platforms").

Another set of hypotheses could be related to the marginal improvement in model forecasts over existing models. As the authors correctly note, a number of platforms exist to provide national degree days based on recent weather. (I was a bit surprised to not see any mention of the Spatial Analytic Framework for Advance Risk Information System [SAFARIS] also sponsored by USDA APHIS.) The authors suggest that one distinguishing feature of the current model is the ability to account for variation in response to degree day accumulations. Yet, no data are provided to measure how the forecast is improved when such variability is added.

- **<u>Response</u>:** We are not sure which "existing models" the reviewer is referring to here. As noted previously, there are no comparable models to our DDRP models for *E. postvittana* and *N. elegantalis*. SAFARIS and USA National Phenology Network host degree-day lookup tables specific to a single species, but most other platforms have only generic degree-day calculators. We have included some information about SAFARIS and its relationship to DDRP in our revision, although there is no publication that we can cite. OSU has been funded by CAPS for several years to provide programming code and species models to SAFARIS, so some of their products were designed by us.

  The assumption that a population will have no variation in development is unrealistic, which is why DDRP was designed to accommodate multiple cohorts. It is of course important to calibrate each species model to create a realistic cohort distribution (i.e. the minimum, maximum, and average emergence time), which we attempted to do for both species. As expected, predictions of first spring egg laying by *E. postvittana* based on a single cohort model exhibited greater differences (in days) from the date of first peak spring flight as estimated from trapping data for most years. However, this analysis is not presented because we do not feel that there is strong justification for using the single chosen emergence time.

The authors description of the method of calculating cold and heat stress is vague. Do they use the same fundamental formulas as are used in CLIMEX. Why? Have they independently vetted those indices to determine how well they perform? Further, why do stress indices need to be determined on a daily basis? Again, with no formal comparisons, I am unclear what new insights the model is providing over existing models.

- **<u>Response</u>:** Fully addressing this entire comment would require expanding our already very long paper to even greater lengths, and it would be beyond the scope of this study. However, we have included some additional information that addresses how DDRP's stress accumulation approach (and formula) compares to the one used by CLIMEX (see "Climatic suitability modeling: species data and parameters" in the Methods), which is also summarized in Table 1. In summary, the cold and heat stress thresholds in DDRP are equivalent to the TTCS (cold stress threshold) and THCS (heat stress threshold) parameters in CLIMEX. In both DDRP and CLIMEX, cold and heat stress units begin to accumulate when temperatures fall below (cold) or above (heat) these thresholds. We note that CLIMEX also offers a degree-day approach to calculate cold and heat stress, but this is intended to accumulate metabolic stress, such as when larvae overwinter but feeding is suppressed by low (indirectly lethal) temperatures, resulting in starvation.

  CLIMEX uses a weekly stress accumulation rate (TCHS and THHS) **and** considers consecutive weeks as a multiplicative factor, which results in an exponential accumulation of stress over the year. Conversely, DDRP accumulates hot and cold stress units on a daily basis, so stress accumulation is linear (non-exponential). These differences appear to be minor in using CLIMEX outputs and parameters to support calibration of DDRP parameters, as demonstrated in our case studies. In particular, we find a great deal of spatial concordance in the areas where each platform predicts temperature stress, a finding that would not be expected if methodological differences were significant.

DDRP calculates stress on a daily basis because it uses daily climate data. Daily data would reflect more fully the underlying variability in temperatures experienced by the organism than averaged weekly data. We assume that CLIMEX calculates stress indices on a weekly basis because it incorporates these estimates into calculations of the Ecoclimatic Index, which also considers the **weekly** growth rate of a population. As described in the Methods, DDRP was designed to model climatic suitability based on daily current or forecast temperature data at fine spatial scales, and CLIMEX is incapable of doing this. We therefore cannot “formally compare” models of the two programs. Rather, we are using CLIMEX as a tool to facilitate DDRP climatic suitability model parameterization.

This model was developed with support from the Cooperative Agricultural Pest Survey. Can the authors describe how surveys were conducted before the development of this platform? How have they changed in response? Is there any evidence that trapping is now more efficient?

- **Response:** DDRP is rather new and still in development. We therefore have no data on its adoption and impacts on CAPS’s activities. This info would require an internal effort conducted by APHIS and is beyond our ability as University scientists.

Current conclusions are not based on the results of the study. They are primarily aspirational.

- **Response:** We disagree with this assessment. As previously noted, the primary purpose of our manuscript is to introduce DDRP as a new decision support tool and illustrate its potential by modeling two focal species that threaten U.S. agriculture. Thus, the majority of our Discussion focuses on 1) what DDRP has to offer and how it is unique from other currently available platforms; 2) the platform’s limitations and potential sources of uncertainty and error in the modeling process; and 3) and interpretation of model products for the two focal species, although we focus on this to a lesser extent because it is not the primary purpose of the study.

RESPONSE TO REVIEWER 2

Reviewer #2: This article describes a new multi-species spatial modeling platform that integrates mapping of phenology and climatic suitability in real-time which has applications for invasive pest detection and management. While many insect phenology modeling platforms are currently available, none to my knowledge, consider that multiple insects cohorts emerging at different times.

Overall, the manuscript is well written. The authors provide good justification for their modeling parameters and adequately discuss the limitations of DDRP. The authors develop two solid insect pest model examples in DDRP which highlight the platform's products and potential as a decision support tool. The modeling is sound, and the R code is readily available online. There is very little that I can fault here. This article is certainly suitable for publication in Plos One.

General Comments:

The manuscript is a bit on the long side. I would recommend some trimming in the discussion section.

- **Response:** We agree that the manuscript is long; however, the relative length of the Discussion is short compared to the Methods. We have therefore tried our best to put extraneous descriptions of methods and analyses in online appendices. Nonetheless, we would be willing to pare down the Discussion if the reviewer has specific suggestions.

Line by Line comments:

143: Replace “ have simulated” with “simulate”

- **Response:** We made this correction.

171: Replace “develop” with “development”

- **Response:** We made this correction.

226: Replace “is” with “in”

- **Response:** We made this correction.

400-405: DDRP seems like it would have a steep learning curve given that it runs best on a Linux OS and requires knowledge of R. How are these forecasting maps going to made available to pest managers and growers planning their pest management schedule for the growing season?

- **Response:** DDRP may be used without knowledge of R as long as the correct libraries are installed and the user understands how to run the script from the command line. However, we agree that customizing code or troubleshooting issues would be difficult for someone who lacks R coding experience. We have written a program user guide for DDRP that is now posted with DDRP files at Zenodo and GitHub (see https://github.com/bbarker505/ddrp_v2/blob/master/DDRP_user_guide_and_platform_requirements_V3.pdf), which should help users learn how to use DDRP. Additionally, we have made some adjustments to DDRP so that it may now run more efficiently on Windows (see "System and software requirements" section of the manuscript for more details).

  We have had several calls with CAPS to get their feedback on interpreting DDRP's products and improving a home page for several CAPS priority species (http://uspest.org/CAPS/). We have also met with SAFARIS to describe and share the DDRP platform and its products.

6. PLOS authors have the option to publish the peer review history of their article (what does this mean?). If published, this will include your full peer review and any attached files.

If you choose "no", your identity will remain anonymous but your review may still be made public.

Do you want your identity to be public for this peer review? For information about this choice, including consent withdrawal, please see our Privacy Policy.

Reviewer #1: No

Reviewer #2: No
